# Switching from Aflibercept to Faricimab in the Treatment of Neovascular Age-Related Macular Degeneration: Short-Term Results from Real-Life Study

**DOI:** 10.3390/jcm14207345

**Published:** 2025-10-17

**Authors:** Jan Kucharczuk, Hubert Kasprzak, Maciej Gawęcki

**Affiliations:** 1 Department of Ophthalmology, 10th Military Clinical Hospital with Polyclinic, 85-681 Bydgoszcz, Poland; jankucharczuk@wp.pl (J.K.); hubert@kasprzak.pro (H.K.); 2 Dobry Wzrok Ophthalmological Center, Szczecinska 23, 80-392 Gdansk, Poland

**Keywords:** faricimab, neovascular AMD, switch, treatment interval, subretinal fluid, intraretinal fluid

## Abstract

**Purpose:** To evaluate anatomical and functional outcomes after switching from aflibercept to faricimab in patients with neovascular age-related macular degeneration (nAMD) with suboptimal response. **Methods:** This retrospective study included 72 eyes of 66 patients with nAMD previously treated with intravitreal aflibercept using a treat-and-extend regimen. Indications for switching included persistent retinal fluid, pigment epithelial detachment (PED), lack of best-corrected visual acuity (BCVA) improvement, or inability to extend treatment intervals beyond four weeks. Patients received three monthly loading doses of faricimab followed by individualized 8- to 16-week dosing. Follow-up comprised six visits over a mean of 8.5 ± 1.4 months. Outcomes included BCVA (logMAR), retinal morphology (subretinal fluid—SRF; intraretinal fluid—IRF; pigment epithelial detachment—PED), central subfoveal thickness (CST), and treatment interval changes. **Results:** Switching to faricimab led to significant short-term anatomical improvement, primarily reduction in subretinal fluid (*p* < 0.0001), with maximal effect during the loading phase. Resolution of SRF was significant at the end of the follow up; however, IRF changes were transient and not sustained beyond three months. PED reduction reached borderline significance (*p* = 0.0455). CST decreased during the loading phase (*p* < 0.0001) but returned to baseline thereafter. BCVA improved only after loading (*p* = 0.0287) but not at final follow-up. Treatment intervals were extended by a mean of ~2 weeks (*p* < 0.0001), increasing in 80% of eyes. Eyes with fewer prior injections and better baseline BCVA achieved superior final visual outcomes. **Conclusions:** Switching to faricimab provides short-term anatomical benefits and treatment-interval extension without sustained visual gain. Functional improvements tended to be greater in patients with fewer injections and shorter treatment duration prior to switch.

## 1. Introduction

Change in medication during intravitreal treatment for neovascular age-related macular degeneration (nAMD) has been widely investigated. A switch of therapy is typically considered in eyes showing inadequate response to a given drug or demonstrating suboptimal morphological or functional outcomes [1,2]. The primary objective of switching is to achieve gains in best-corrected visual acuity (BCVA) and improvements in retinal morphology.

An additional potential benefit of switching—highlighted in recent studies—is the extension of treatment intervals, which can reduce treatment burden and improve patients’ quality of life [3,4]. This is particularly relevant given the well-established understanding that therapy for nAMD is usually lifelong.

Whenever a new intravitreal medication is introduced, its efficacy is evaluated not only as a first-line treatment but also as an alternative for patients already undergoing therapy. Numerous studies have demonstrated significant benefits when switching patients from off-label bevacizumab or ranibizumab to aflibercept, which subsequently became the most widely used approved medication for nAMD over the past decade [5,6].

With the recent introduction of faricimab, there is a need for contemporary analyses assessing its efficacy in patients with insufficient response to aflibercept. The aim of our study was to evaluate the outcomes of switching from aflibercept to faricimab in patients with nAMD, focusing on changes in BCVA, retinal morphology, and treatment interval extension.

## 2. Material and Methods

The study adhered to the tenets of the Declaration of Helsinki. Institutional review board approval for the retrospective analysis was obtained (KB-1042/2025), and all patients provided informed consent for participation in the treatment program.

The study cohort comprised 72 eyes of 66 patients treated with intravitreal injections for neovascular age-related macular degeneration (nAMD) at the Department of Ophthalmology of the 10 Military University Hospital in Bydgoszcz, Poland. All treatments were performed in accordance with the Polish National Programme for AMD therapy, which follows the product characteristics of each approved drug.

All patients had previously received 2 mg intravitreal aflibercept using a treat-and-extend regimen but showed suboptimal response. Under these circumstances, therapy was switched to faricimab (Vabysmo). Indications for the switch included at least one of the following:

- Persistent subretinal fluid (SRF), intraretinal fluid (IRF), or pigment epithelial detachment (PED).
- Subretinal hemorrhage or subretinal hyperreflective material (SHRM) persistent after injection.
- No improvement in best-corrected visual acuity (BCVA).
- Inability to extend the treatment interval beyond four weeks.

Only patients with at least six post-switch follow-up visits were included in the analysis. Treatment with faricimab followed the official updated product characteristics [7]. Three monthly loading doses were administered initially, followed by individualized dosing intervals determined by disease activity with the minimum interval of 8 weeks and maximum of 16 weeks. In eyes demonstrating an optimal response, the interval between injections was progressively extended according to the treat-and-extend protocol described in the product specification. Each eye was evaluated at six post-switch visits—the first three performed monthly and the subsequent three scheduled at 8–16-week intervals, depending on disease activity. Hence, observation time post switch was carried out for mean 8.50 (+/−1.43) months, median 8.13 (quartile: 7.58–9.14). Assessments at every visit included: BCVA measurement using the logMAR chart, slit-lamp examination of the anterior and posterior segments, swept-source OCT (Triton, Topcon Corporation, Tokyo, Japan 2017) (SS-OCT) with quantitative assessment of: central subfoveal retinal thickness (CST), and qualitative evaluation of retinal morphology (presence of SRF, IRF, PED, hemorrhage, or SHRM). IRF degenerative changes, such as retinal tubulations, were not considered a therapeutic target and their persistence was not considered a lack of improvement after switch. The SS-OCT images were evaluated by two independent observers. In cases where the interpretation of the retinal architecture was controversial, a consensus was reached after discussion between the two. Baseline evaluation also included classification of macular neovascularization (MNV) type according to the Spaide classification [8]. Type 4 MNV was determined as mixed type MNV 1 and MNV 2 according to recent report [9]. Polypoidal choroidal vasculopathy (PCV) was recognized according to OCT criteria provided by Asia-Pacific Ocular Imaging Society PCV Workgroup [10]. Fluorescein angiography was performed in every subject at baseline in the diagnostic process. The final analysis considered both anatomical and functional outcomes as well as the treatment interval achieved during therapy.

### Statistical Procedures

Categorical variables were presented as absolute numbers and percentages (frequencies). Numerical variables were summarized using the mean, median, standard deviation, and interquartile range (lower–upper quartile). The normality of data distribution was assessed with the Shapiro–Wilk test, and the Levene test was applied to verify homogeneity of variances.

For normally distributed variables, a multifactor repeated-measures analysis of variance (ANOVA) was used to evaluate temporal changes. Mauchly’s sphericity test was applied to confirm the validity of the repeated-measures ANOVA. For non-normally distributed data, generalized estimating equations (GEE) were employed. All multivariate models were adjusted for age, sex, baseline treatment interval, and prior treatment duration.

Relationships between numerical variables were analyzed using Spearman’s rank correlation coefficients. A *p*-value of <0.05 was considered statistically significant. All analyses were performed using Statistica™ software, version 13.3 (TIBCO Software Inc., Palo Alto, CA, USA).

## 3. Results

Baseline demographic characteristics of the study cohort, including treatment duration and number of received intravitreal treatments is provided in Table 1.

**Figure 1:**
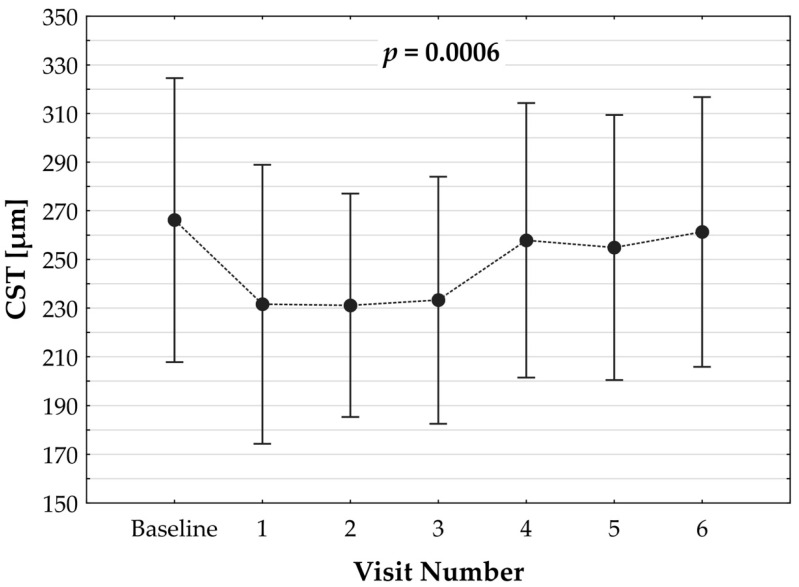
Dynamics of changes in central subfoveal thickness (CST) over the observation period post switch. *p* value refers to the statistical model.

**Figure 2:**
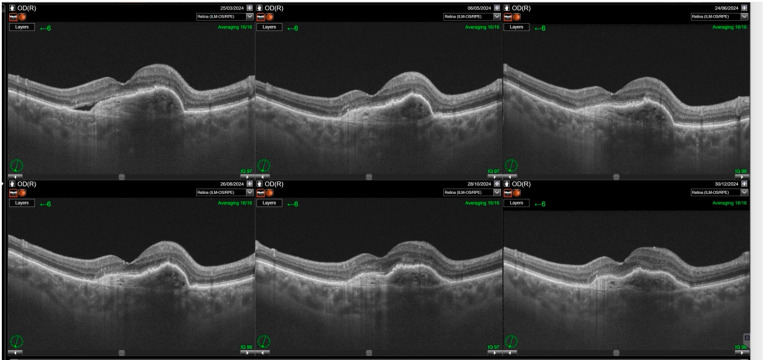
Significant improvements in retinal morphology noted through the follow-up. Complete resolution of subretinal fluid (SRF) and intraretinal retinal fluid (IRF) is noted through the whole observation time post first injection.

**Figure 3:**
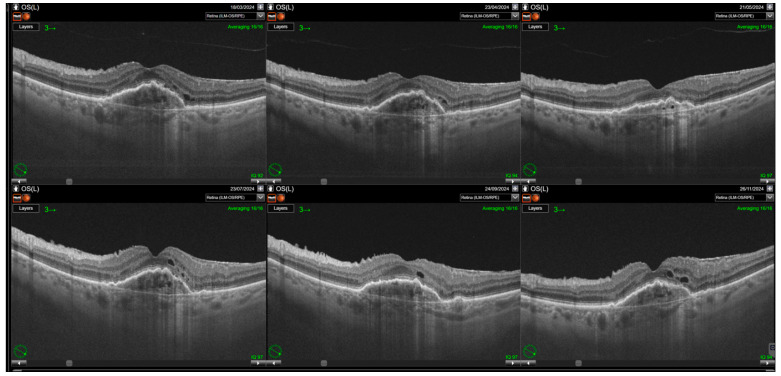
Relapse of intraretinal fluid after the loading phase. Resolution of intraretinal fluid (IRF) is achieved following the loading phase of treatment; however, IRF reappears with the extension of treatment intervals. Some cystic spaces visible on the scans correspond to neurodegenerative changes (collared, round retinal tubulations). Nevertheless, a significant increase in small intraretinal cystic lesions reflecting disease activity is also observed.

**Figure 4:**
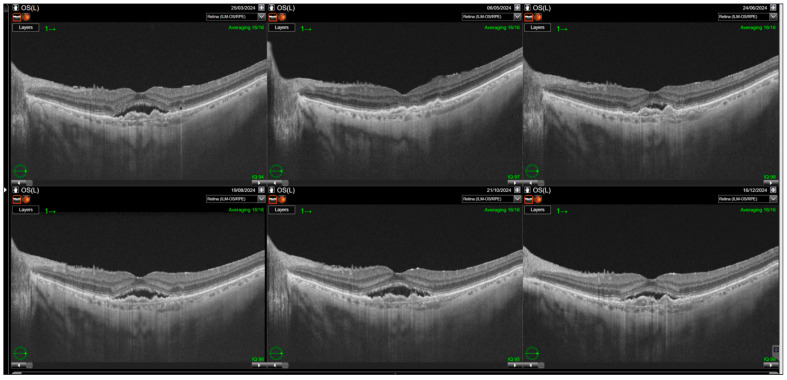
Relapse of subretinal fluid noted after the loading phase. Resolution of the subretinal fluid (SRF) is observed after the first injection with gradual increase in its amount during the rest of the follow-up.

Switching to faricimab in the treatment of nAMD produced anatomical improvements that followed a distinct temporal pattern during post-switch follow-up. By the end of the observation period, retinal morphology demonstrated clear improvement, driven primarily by a reduction in SRF. In contrast, changes in IRF were not statistically significant, while reductions in PED reached borderline statistical significance at the final visit (*p* = 0.0455). The greatest morphological benefit was observed at visits 2 and 3, corresponding to the second phase of the loading regimen, during which the reduction in residual IRF was also statistically meaningful. However, as treatment intervals were subsequently extended, the magnitude of these anatomical gains gradually declined (Table 2). Loss of early improvement after the loading phase was also evident in SS-OCT parameters, specifically CST (Table 3; Figure 1). Retinal thickness showed a significant reduction compared with baseline during the loading phase, but this benefit gradually waned and was no longer present at the end of follow-up. Examples of SS-OCT scans at baseline and through the follow-up are presented in Figure 2 (good response) and Figure 3 and Figure 4 (poor response).

Switching to faricimab did not lead to sustained functional improvement. A significant gain in BCVA was noted only at a single time point—after completion of the loading phase—but not at the final follow-up (Table 4). Nevertheless, switching to Vabysmo allowed for a significant extension of the treatment interval by approximately 2 weeks (Table 5). The treatment interval increased in 80.28% of eyes, remained unchanged in 18.31%, and decreased in only one eye (1.41%).

Analysis of the relationship between baseline factors and final outcomes using generalized linear models (GLZ) yielded several significant findings. The presented β values represent correlation coefficients obtained through multivariate regression with robust standard errors to account for deviations from normality.

### 3.1. Best-Corrected Visual Acuity (BCVA)

BCVA at 6 months was significantly associated with baseline BCVA (β = 0.72, *p* < 0.0001) and with the number of injections prior to the switch (β = 0.23, *p* = 0.0021). Eyes with better baseline visual acuity and fewer prior injections achieved better BCVA at 6th follow-up visit. In contrast, the relationship between BCVA and the last treatment interval before the switch was not statistically significant (*p* = 0.1320).

### 3.2. Central Subfoveal Retinal Thickness (CST)

CST at 6 months was significantly associated with baseline CST (β = 0.53, *p* < 0.0001), with higher baseline CST predicting higher CST at follow-up. In addition, CST at 6th visit was dependent on the last treatment interval prior to the switch (β = –0.23, *p* = 0.0162), indicating that longer intervals were associated with lower CST at follow-up.

## 4. Discussion

Switch to faricimab from aflibercept in patients with suboptimal response in the treatment of nAMD provides moderate morphological improvements and two-week extension of treatment interval. However, no significant BCVA gains were observed after approximately eight months of post switch follow-up.

Most patients demonstrated a characteristic pattern of response following the switch. During the initial loading phase, marked reductions in SRF, IRF, and CST were observed. These early anatomical responses were followed by partial loss of effect as the treatment interval was extended, while BCVA remained largely stable at pre-switch levels. Nevertheless, a meaningful proportion of patients achieved complete SRF resolution, though IRF tended to persist. The ability to achieve subretinal fluid resolution while extending the treatment interval in nearly 80% of eyes can be regarded as a therapeutic success in this treatment-resistant population.

The resolution of SRF in a substantial subset of patients is clinically important. SRF is widely recognized as a hallmark of MNV activity and a biomarker of treatment efficacy. The observed SRF reduction, despite prior resistance to aflibercept, suggests that faricimab may offer an additional mechanism of action relevant to disease control. Conversely, IRF fluctuations require separate consideration. Persistent or recurrent IRF is strongly associated with photoreceptor damage, fibrosis, and RPE atrophy, leading to progressive visual decline and suboptimal BCVA outcomes despite continued therapy [11,12,13]. Previous studies have shown that IRF persistence correlates with poorer long-term visual prognosis, whereas SRF, though an indicator of active disease, may have a less detrimental or even protective effect on vision in some cases [14,15,16,17,18].

In our cohort, IRF reduction was predominantly observed during the loading phase, followed by reaccumulation once the dosing interval exceeded four weeks. CST changes followed a similar temporal pattern, with initial improvement and subsequent return to near-baseline values. This pattern suggests that while faricimab is effective in achieving short-term morphological improvements, the durability of these effects diminishes as treatment intervals are extended. These findings are consistent with the chronic course of nAMD, in which long-standing neurodegenerative changes in the outer retina limit functional recovery, even when exudation is controlled.

A transient BCVA increase was recorded at the end of the loading phase, but visual acuity later stabilized at pre-switch levels. This plateau likely reflects a ceiling effect, as most patients in this study had relatively good baseline vision at the time of switching. In eyes with limited visual potential due to chronic structural changes, functional improvement is less likely to occur despite apparent anatomical gains.

Correlation analyses between baseline characteristics and final outcomes provided additional insight. On average, patients received 32 intravitreal injections over approximately 52 months before switching to faricimab. Eyes that had undergone fewer prior injections and had a shorter duration of anti-VEGF exposure achieved better final BCVA, suggesting that earlier intervention with faricimab may yield superior results in suboptimal responders. Similarly, eyes with higher baseline BCVA tended to maintain better vision during follow-up. These findings support the rationale for earlier therapeutic switching in cases of partial or waning response, rather than delaying until extensive retinal remodeling has occurred.

The relationship between treatment history and post-switch outcomes also warrants consideration. Among patients with poor response before switching, those who maintained longer treatment intervals demonstrated more favorable anatomical improvement after the switch, reflected by lower CST values. This may indicate that eyes capable of sustaining disease control at extended intervals before switching retain a degree of retinal integrity, allowing for improved responsiveness to a new therapeutic agent. In such cases, switching to faricimab might enhance durability while maintaining morphological stability. However, the decision to switch should always consider the persistence of fluid, the presence of outer retinal atrophy, and the potential for meaningful visual or anatomical recovery.

The outcomes observed in our cohort align with findings from other published studies investigating faricimab as a switch therapy. Eckardt et al. reported only moderate improvements in retinal and choroidal morphology in patients previously treated with ranibizumab or aflibercept [19]. Liu et al. demonstrated significant short-term anatomical gains—including reductions in CST, PED, IRF, and SRF—one month after switching, though without functional benefit [20]. Schneider et al. observed comparable results, noting anatomical improvement but no BCVA change over short-term follow-up [21]. In a one-year study, Grimaldi et al. found that faricimab converted a substantial proportion of MNV lesions from active to inactive (39% to 63%), while allowing extension of treatment intervals and maintenance of visual acuity [22]. Takahashi et al. also documented decreased choroidal thickness and longer dosing intervals after one year, with stable BCVA [23]. Similarly, other real-world series have reported consistent anatomical improvement and interval extension, albeit without visual gain [24,25,26].

Collectively, the current evidence supports a coherent conclusion: switching to faricimab offers structural and practical benefits—reductions in retinal fluid, thinning of the macula, and longer retreatment intervals—but does not typically improve visual function. The dual inhibition of angiopoietin-2 and VEGF-A may provide additional vascular stabilization in previously refractory eyes, yet irreversible photoreceptor and RPE damage likely limit the potential for visual recovery. Importantly, treatment-interval extension reduces patient and caregiver burden, visit frequency, and healthcare resource use, representing a clinically meaningful outcome in chronic disease management.

However, the persistence of IRF and the lack of sustained CST reduction highlight the need for individualized dosing regimens and careful monitoring. Overly aggressive extension of treatment intervals may lead to reaccumulation of fluid and potential deterioration of retinal integrity. Some authors have even argued that maintaining a regular anti-VEGF regimen may yield superior long-term visual outcomes and quality of life compared with repeated switching or aggressive extension strategies [27]. Therefore, the decision to switch or extend should balance anatomical benefit, functional stability, and patient burden.

## 5. Study Limitations

The principal limitations of this study include its relatively short observation period and moderate sample size. Longer follow-up and larger multicenter cohorts are needed to evaluate the long-term anatomical durability and functional implications of switching to faricimab in nAMD. In addition, variability in treatment history and dosing intervals among patients may have influenced the outcomes.

## 6. Conclusions

Switching to faricimab in patients with nAMD reduces the persistence SRF and PED but has limited effect on IRF under standard dosing regimens. No significant improvement in BCVA was observed. On average, the switch allowed for an extension of the treatment interval by approximately two weeks. Shorter prior treatment duration and fewer intravitreal injections before the switch were associated with better functional outcomes.

## Figures and Tables

**Table 1:** Baseline characteristics of the study cohort.

Analyzed Trait	Values
No. of participants, n	66
No. of eyes, n	72
Gender, n (%)	Female: 33 (50.0%)
	Male: 33 (50.0%)
Age [y], M (SD)	76.51 (6.56)
MNV type, n (%)	Type 1: 62 (86.11%)
	Type 2: 1 (1.39%)
	Type 3: 5 (6.94%)
	Type 4: 4 (5.56%)
PCV, n (%)	4 (5.56%)
SRF, n (%)	61 (84.72%)
IRF, n (%)	23 (31.94%)
SHRM, n (%)	9 (12.50%)
Hemorrhage, n (%)	4 (5.56%)
PED, n (%)	12 (16.67%)
CST [µm], M (SD)	266.19 (58.37)
Visual acuity [logMAR], M (SD)	0.25 (0.22)
No. of injections before switching, M (SD)	32.49 (19.29)
Last interval duration [w], M (SD)	5.67 (1.93)
Treatment duration before switch [m], M (SD)	52.89 (37.60)
Overall treatment duration [m], M (SD)	63.25 (38.10)

n—number; %—percentage; M—mean; SD—standard deviation; BCVA—best corrected visual acuity; logMAR—logarithm of the minimum angle of resolution; CST—central subfoveal retinal thickness; PED—pigment epithelial detachment; MNV—macular neovascular membranes; PCV—polypoidal choroidal vasculopathy; SRF—subretinal fluid; IRF—intraretinal fluid; SHRM—subretinal hyperreflective material; m—months.

**Table 2:** Prevalence of investigated retinal abnormalities over the course of the study (*n* = 72).

Analyzed Condition	Baseline	Visit 1	Visit 2	Visit 3	Visit 4	Visit 5	Visit 6	*p*-Value *
SRF	60 (84.51%)	23 (31.94%)	20 (28.17%)	21 (29.17%)	37 (51.39%)	35 (49.30%)	37 (52.11%)	<0.0001
IRF	23 (31.94%)	9 (12.50%)	9 (12.50%)	9 (12.50%)	20 (27.78%)	22 (30.99%)	20 (28.17%)	0.3657
SHRM	9 (12.50%)	4 (5.56%)	4 (5.56%)	3 (4.17%)	5 (6.94%)	4 (5.63%)	3 (4.22%)	0.1317
Hemorrhage	4 (5.56%)	3 (4.17%)	2 (2.78%)	1 (1.39%)	2 (2.78%)	0 (0.00%)	1 (1.41%)	0.0833
PED	12 (16.67%)	9 (12.50%)	8 (11.11%)	9 (12.50%)	9 (12.50%)	8 (11.27%)	8 (11.27%)	0.0455

* Statistical significance levels for differences between baseline and last visits, n—number; %—percentage; M—mean; SD—standard deviation; PED—pigment epithelial detachment; SRF—subretinal fluid; IRF—intraretinal fluid; SHRM—subretinal hyperreflective material. Missing data were pairwise deleted.

**Table 3:** The observed dynamics of CST [µm] in the studied eyes after switch (*n* = 72).

Visit	M	SD	Omnibus *p* Value	Paired *p* Value *
Baseline	266.19	58.37	0.0006	–
1	231.65	57.28		<0.0001
2	231.17	45.88		<0.0001
3	233.29	50.73		<0.0001
4	257.90	56.38		0.3248
5	254.90	54.47		0.0923
6	261.35	55.41		0.3987

* M—mean; SD—standard deviation; CST—central subfoveal retinal thickness. The first *p* value (Omnibus) refers to the overall model; the right-hand *p* values (Paired) concern pairwise comparisons with baseline.

**Table 4:** The observed dynamics of BCVA [logMAR] in the studied eyes after switch (*n* = 72).

Visit	M	SD	Omnibus *p* Value	Paired *p* Value *
Baseline	0.25	0.22	0.0002	–
1	0.24	0.20		0.2739
2	0.23	0.24		0.1698
3	0.22	0.20		0.0287
4	0.24	0.20		0.5453
5	0.27	0.25		0.3367
6	0.28	0.24		0.1001

* M—mean; SD—standard deviation; BCVA—best corrected visual acuity; logMAR—logarithm of the minimum angle of resolution. The first *p* value (Omnibus) refers to the overall model; the right-hand *p* values (paired) concern pairwise comparisons with baseline.

**Table 5:** Treatment intervals [weeks] in the studied patients (*n* = 72).

Visit	M	SD	*p* Value
Baseline	5.67	1.93	<0.0001
Final (6) post switch	8.86	2.25	–
Delta	3.07	2.43	–

M—mean; SD—standard deviation.

## Data Availability

The original contributions presented in the study are included in the article. Further inquiries can be directed to the corresponding author.
